# A roadmap for a middleware as a federation service for integrative data retrieval of agricultural data

**DOI:** 10.1515/jib-2024-0027

**Published:** 2024-11-07

**Authors:** Jorge García Brizuela, Carsten Scharfenberg, Carmen Scheuner, Florian Hoedt, Patrick König, Angela Kranz, Antonia Leidel, Daniel Martini, Gabriel Schneider, Julian Schneider, Lea Sophie Singson, Harald von Waldow, Nils Wehrmeyer, Björn Usadel, Stephan Lesch, Xenia Specka, Matthias Lange, Daniel Arend

**Affiliations:** Leibniz Institute of Plant Genetics and Crop Plant Research (IPK), D-06466 Gatersleben, Germany, https://www.ipk-gatersleben.de/; Leibniz Centre for Agricultural Landscape Research (ZALF), D-15374 Müncheberg, Germany, https://www.zalf.de/; Senckenberg Museum of Natural History Görlitz, D-02826 Görlitz, Germany, https://museumgoerlitz.senckenberg.de/; Johann Heinrich von Thünen-Institut, D-38116 Braunschweig, Germany, https://www.thuenen.de/; Forschungszentrum Jülich GmbH (FZJ), IBG-4, D-52428 Jülich, Germany, https://www.fz-juelich.de/en; Kuratorium für Technik und Bauwesen in der Landwirtschaft (KTBL), D-64289 Darmstadt, Germany, https://www.ktbl.de/; ZB MED – Information Centre for Life Sciences, D-50931 Cologne, Germany, https://www.zbmed.de/; Leibniz-institute for Information Infrastructure (FIZ Karlsruhe), D-76344 Karlsruhe, Germany, https://www.fiz-karlsruhe.de/

**Keywords:** FAIRagro, federate research data infrastructures, middleware, NFDI

## Abstract

Agriculture is confronted with several challenges such as climate change, the loss of biodiversity and stagnating productivity. The massive increasing amount of data and new digital technologies promise to overcome them, but they necessitate careful data integration and data management to make them usable. The FAIRagro consortium is part of the National Research Data Infrastructure (NFDI) in Germany and will develop FAIR compliant infrastructure services for the agrosystems science community, which will be integrated in the existing research data infrastructure service landscape. Here we present the initial steps of designing and implementing the FAIRagro middleware infrastructure to connect existing data infrastructures. The middleware will feature services for the seamless data integration across diverse infrastructures. Data and metadata are streamlined for research in agrosystems science by downstream processing in the central FAIRagro Search and Inventory Portal and the data integration and analysis workflow system “SciWIn”.

## 1 Introduction

Agriculture is facing increasing challenges, such as growing food demand but stagnating productivity, climate change, biodiversity loss and degradation of natural resources [1, 2]. Data and digital technologies offer the potential to support the ambitious goal to overcome these challenges. Agricultural sciences are an interdisciplinary field ranging from genetics and molecular biology to crop, soil, animal and forest sciences, agro- and geoecology, agrotechnology, market economics and social and cultural sciences. This range of disciplines and technologies offers the opportunity to systematically study as many potentially relevant contributing factors as possible to understand their complex interactions across scales. The reciprocal relationships between the environment, landscape, climatic conditions and human land use need to be better understood. As we have entered the era of “big data”, the tremendous increase in recorded measurement data creates new challenges and opportunities. In addition to the sheer volume of data that needs to be stored securely and efficiently, the challenges also include the large heterogeneity of the data relevant to agricultural science, which require elaborate but also reproducible ontology alignment, schema mapping, data integration and data fusion 3], [4], [5. Integrated and fused data sets offer the opportunity to make complex patterns and statistical relationships in the data visible using techniques such as machine learning and deep learning [6].

The National Research Data Infrastructure (NFDI) e. V. association [7] was founded jointly by the federal government and all federal states in 2020 to establish coordinated research data management in Germany [8]. The vision is to make the mostly decentralized, project-related and time-limited available data accessible as a common good for science and society according to FAIR principles [9]. The individual scientific disciplines are collaborating in different NFDI consortia. The FAIRagro consortium [10, 11] collaboratively implements infrastructure services for a federated, interoperable, scalable and sustainable research data infrastructure (RDI) in the agrosystems science community and integrates them into the national and international RDI service landscape. These infrastructure services are responsible for interconnecting existing domain-specific or institutional RDIs to enable the accessibility and usability of research data in accordance with the FAIR data principles [9]. Members of the FAIRagro consortium provide a broad set of RDIs for different agrosystems science disciplines [12], which are based on diverse technological infrastructures and established different organizational processes. This makes a tight interlinking between them challenging and requires a more loosely coupled federation of autonomous RDIs. Therefore, one key objective of FAIRagro is the development of an infrastructure component acting as middleware, which connects all RDIs and integrates their provided datasets, but guarantees their operational, organizational and technical autonomy. This middleware will be a central component and embedded into the wider FAIRagro ecosystem to implement essential services for a federated and FAIR-compliant research data management (RDM).

In this article we explain the FAIRagro middleware concept and its implementation approach along with various considerations and concepts that were taken into account in the design of the middleware.

## 2 Synthesis of the federated FAIRagro middleware architecture

Presently, agrosystems research data infrastructures lack standardized application programming interfaces (API), hindering the findability, accessibility and reusability of datasets. FAIRagro aims to network existing infrastructures in order to increase the compliance of published datasets with the FAIR criteria. To achieve interoperability among diverse disciplinary and technical systems while keeping the technical and organizational autonomy of existing infrastructure intact, a decision about the fundamental architecture of the service ensuring interoperability was necessary.

On one hand, there was the consideration of establishing a large central system (e.g. GeRDI [13]) where infrastructural connections are facilitated through individual microservices. In this scenario, the bulk of the effort for achieving interoperability would be centralized within the development of this central system. However, this would make the integration of additional infrastructures in the future more cumbersome. To address the requirement of an infrastructure autonomy, a more lightweight and decentralized, i.e. federated, concept was in favor, involving the development of a streamlined service defining standardized exchange formats for metadata and data exchange. These services, facilitating communication between infrastructure and downstream data processing services, are subsumed under the term “middleware”. With this approach, the implementation effort would be distributed, as the infrastructure operators could implement the connection to the middleware individually based on the standardized API of the middleware (e.g. BrAPI [14]).

The concept of a middleware framework linking various RDIs in agrosystem sciences (see Section 2.1) was developed by FAIRagro partners beginning in 2019. Since then, the middleware concept went through a sharpening and consolidation process. The initial idea was based on the approach of specifying harmonized application programming interfaces (APIs), as well as implementing the API for all participating RDIs. The API specification and architecture concept were based on technical-functional and operational requirements analysis from scientific use cases and downstream services, such as a Search and Inventory Portal or a Workflow System Infrastructure for distributed data analysis. On this basis, ETL (Extract-Transform-Load) pipelines [15] had to be implemented to provide quality-assured, so-called FAIR data containers in a data warehouse-like infrastructure. Today, we refer to this as the FAIR Digital Objects (FDO) concept [16]. This first concept was assessed as not sufficiently scalable against the background of the broad range of agricultural sciences. In particular, the number of data providers to be considered, the complexity of the data access patterns for science, the high permanent maintenance effort of the API implementations, as well as the interference with autonomy and insufficient concepts for handling sensitive data were major obstacles to acceptance.

The continuous exchange with researchers in the agrosystems sciences and consolidation processes led to the revision of the concept in 2020, resulting in an API concept that was supposed to primarily implement harmonized metadata access. The actual data access was to be provided via a metadata Search and Inventory Portal to the original data access interfaces provided by the data providers. The middleware should provide a service for the integrative retrieval of Schema.org [17] compliant metadata, which was to be enriched with information on the specific data access. Although barriers of the first concept were thereby tackled, the broad range of data domains to be addressed and thus the data and metadata types to be integrated was too large. This concept was also unable to resolve the issue of access to sensitive data. Therefore, the concept to be developed should combine the advantages of federated infrastructures, FDOs, metadata harvesting and mapping to common metadata standards, as well as granular data access authorization and implement this using three prioritized agricultural domains soil, plant and environment as a nucleus for later extensions.

### 2.1 Landscape analysis

FAIRagro partners provide 13 different RDIs across various disciplines in the agrosystem sciences that will be connected via the FAIRagro middleware [12]. These RDIs have a different technological basis, apply different metadata standards or APIs which makes a tight interlinking between them challenging. We decided upon an iterative approach consisting of three phases to mitigate the complexity of this task. In the first phase a set of five RDIs will be connected and the general concept of the middleware will be developed. In the second phase, additional infrastructures will be integrated to complement the interoperable RDI network, addressing key areas of the agrosystems community. In the third phase, infrastructures of other NFDI consortia will be integrated to enable cross-cutting, interdisciplinary exchange of (meta-)data.

In the initial phase, our approach started with an analysis of the key characteristics of the first five infrastructures to be connected by the middleware. The analysis drew upon available information coming from reports, research papers or technical documentation of the underlying technological system of the infrastructures. The analysis encompassed the following aspects: data domain, use of persistent identifiers (PIDs), metadata standards, metadata serialization formats, available APIs, for metadata or data access, used technologies, licenses, accessibility and references. But this literature-based analysis uncovered missing information not readily apparent in the initial sources. Therefore, we also had direct exchanges and interviews with the infrastructure operators to gain a better understanding of the infrastructures and to obtain missing information complementing the literature analysis.

Table 1 shows the result of the literature analysis and direct exchange with infrastructure operators. The infrastructures encompass different domains in agrosystems science, including genotypic and phenotypic data, soil studies, and forestry research. Every RDI is based on a different technology stack. With respect to metadata there are some similarities as all the infrastructures offer basic metadata based on the DublinCore (DC) [26]. With the exception of the Thünen Atlas, all RDIs provide Digital Object Identifiers (DOIs) for their datasets and can export their metadata in a JSON format. Three of them are compliant with Schema.org. Notably, all platforms leverage standardized metadata and structured data formatting, facilitating data integration and exchange. In general, all RDIs provide open accessible datasets, but three of them also share some restricted datasets, which can be only accessed on request. A license on the data that allows open use but requires citing the dataset is a common requirement. But the licenses may vary for single datasets. Also, the texts of the licenses vary between the infrastructures. They can be clustered into the categories public domain or unrestricted re-use (PD), attribution required (BY), attribution required and commercial re-use forbidden (BY, NC). The dominating service used for licenses are the Creative Commons Licenses [41]. However, since there are legal challenges when it comes to the protection of research data under the copyright act, we chose an abstract description of the data without citing the specific license used. Most of the datasets are freely accessible with only some datasets being restricted. That being said: the metadata is always available even for the restricted data sets.

**Table 1:** Summary of main characteristics of FAIRagro’s core research data infrastructures: e!DAL-PGP, BonaRes repository, OpenAgrar, PUBLISSO and Thünen Atlas. License explanation: PD = public domain, BY = open to use but cite as source, NC = open for noncommercial use when citing as source; access explanation: OA = open access, Req = data only available upon request.

Evaluated aspects	e!DAL-PGP [18, 19]	BonaRes repository [20, 21]	OpenAgrar [22]	PUBLISSO [23]	Thünen Atlas [24]
Data domain	Genomic and phenomic	Soil, agriculture, ecology and geodata	Agriculture	Life science	Agriculture, forestry, fisheries, rural studies
Persistent identifier	DOI [25]	DOI [25]	DOI [25]	DOI [25]	–
Metadata standards	DC [26]	DC [26], INSPIRE [27], DataCite [28]	DC [26], MODS [29]	DC [26], MODS [29]	DC [26], INSPIRE [27], DIF [30]
Metadata serialization format	Schema.org (JSON-LD) [17], Bioschemas (JSON-LD) [31]	Schema.org (JSON-LD) [17]	Schema.org (JSON-LD) [17]	JSON-LD	JSON, XML
API type	OAI-PMH [32]	OGC [33]	REST [34], OAI-PMH [32]	REST [34], OAI-PMH [32]	REST [34], OAI-PMH [32], OGC [33]
Technologies	e!DAL [35]	smart.finder SDI, map.apps [36]	MyCore [37]	to.science [38]	GeoNode [39], GeoServer [40]
License	PD, BY, NC	BY	BY	BY	PD, BY, NC
Access	OA	OA, Req	OA, Req	OA, Req	OA, Req

This analysis was the basis for understanding the similarities and differences across infrastructures and will be the basis for developing the middleware concept.

### 2.2 Requirements

In this section we describe the iterative process for analyzing and defining specific requirements for the middleware infrastructure which originated in the beginning mainly from the FAIRagro proposal [11]. These requirements were concretized based on the specific needs of six initial FAIRagro use cases [42], which require access to research data from different FAIRagro infrastructures. These six use cases are based on scientific questions in the agrosystem sciences addressing certain challenges in the areas of research data management. To identify the concrete requirements of the use cases with respect to the middleware we initiated a three-stage on-boarding process between the use case leads and FAIRagro members involved in the middleware development. The latter initiated the process by organizing an online meeting to introduce the objectives of RDM services to be developed. In a second step, there was a comprehensive virtual on-boarding workshop organized by the use case leads to explain their actual needs and give a detailed introduction into domain-specific challenges and use-case relevant requirements. In the third stage we held an in-person retreat meeting to collect feedback on first ideas of the middleware and to discuss ideas raised during the previous on-boarding events. Based on this, a draft outlining concrete requirement for the middleware and essential interfaces was developed. This draft was refined by collecting additional feedback during several internal FAIRagro community meetings and as well as different cross-NFDI events. As a result of these four major technical requirements were defined and will be described subsequently.

#### 2.2.1 Exchange of metadata

One of the main tasks of the middleware is to act as a bridge between the research data infrastructures (RDIs) and the FAIRagro Search and Inventory Portal, which will build its dataset search functionality upon rich metadata conforming to a schema that will be specifically designed for datasets in the agrosystem community. Thus, the middleware will interface and harvest all RDIs for metadata and persist them. The metadata format has to comply with the requirements of the Search and Inventory Portal.

The Search and Inventory Portal will be based upon the widely-used Dataverse project [43], which has the ability to define custom metadata blocks for datasets to make them search- and facetable. Metadata can be entered in the frontend, ingested as a Dataverse-specific format via its API, or harvested according to the OAI-PMH protocol. Dataverse has a large and active global community, and is already being used for the NFDI4Health [44] German Central Study Hub serving a similar purpose, opening many possibilities for effective collaboration.

#### 2.2.2 Provision of metadata and data for data processing services

FAIRagro will develop a Scientific Workflow Infrastructure (SciWIn) which will provide a platform to manage reproducible computational workflows. SciWIn will be based on FDOs [45], e.g. in the form of RO-Crates [46], to represent data- and code-artifacts along with their metadata and provenance information. The FAIRagro middleware will act as a broker of these RO-Crates and facilitate the exchange of information between SciWIn, data- and code-repositories and the FAIRagro Search and Inventory Portal.

#### 2.2.3 Continuous recording of technical quality metrics

Alongside the Search and Inventory Portal of FAIRagro, an inventory of research data infrastructures is being developed. Its functionality relies on the middleware, which facilitates the communication between infrastructures. This metadata not only describes the datasets but also includes information relevant to their quality and usability. In a future advanced version of the inventory service, it will also include information on the quality and availability of services based on objective metrics (e.g. service downtime and/or “currently service unavailable” and response times) and service summaries (e.g. number of records, size of data, and special visualization capabilities) that can be filtered on. Additionally, simple quantitative indicators related to the number of datasets or sizes will be added to allow users to prioritize infrastructures.

#### 2.2.4 Access to sensitive data and connection to cross-NFDI authentication and authorization infrastructure

Some of the FAIRagro RDIs share restricted datasets, which can only be accessed on request, and provide data with various licensing terms. Thus, the need for an authentication and authorization infrastructure (AAI) system arises to ensure seamless user access across platforms. By connecting to an AAI and thereby adding a user context to the middleware, it is also possible to access restricted datasets both manually by users and automatically. For restricted data, basic bibliographic metadata will be freely accessible in order to make the dataset findable. Furthermore, specific metadata about access restrictions determines the accessibility of a dataset. To enable access to restricted data, the middleware needs to handle respective requests by users, communication between users and providers and access grants for certain datasets. As the middleware is intended to broker access to the SciWIn, single sign-on (SSO) functionality is another requirement. SSO allows users to securely authenticate themselves across multiple applications using just one set of login credentials, i.e. users can login once and access multiple services without having to enter their credentials again. Finally, by connecting to an AAI system, the middleware service can be extended to interconnect FAIRagro infrastructures with the NFDI or international infrastructure networks e.g. ELIXIR [47], enabling cross-disciplinary data sharing. This will involve collaboration with other NFDI consortia to define guidelines for harmonized APIs. The aim is to integrate the FAIRagro infrastructures into the overall NFDI AAI architecture [48] as well as into international networks.

During the initially mentioned process of determining the technical requirements, it became evident that also operational requirements need to be considered. In general, we need a loosely coupled federation of autonomous RDI with no direct intervention of the middleware to the RDIs. The organizational autonomy of every RDI needs to be guaranteed. However, minor changes such as the export of schemas or the implementation of data standards, which do not influence the established data acquisition and data provision process of the RDIs, has to be requested in a guided and collaborative manner. The implementation of these changes is mandated by RDI operational teams under the supervision of hosting institutes with consultation and advice of FAIRagro.

### 2.3 Analysis and evaluation of existing concepts and technical solutions

We evaluated different relevant technologies and concepts that had the potential to influence the development of our middleware approach. In the following section we describe our considerations according to the metadata schema, which can be used for data integration and our investigation of a relevant approach for a suitable AAI that can be integrated into the middleware architecture. To assess the suitability of three potential middleware platforms or software solutions for our needs, we will conduct a comprehensive evaluation that considers their strengths, limitations, potential benefits in our context, and any external factors that could impact their success.

#### 2.3.1 Data integration & metadata schema

When analyzing the FAIRagro infrastructures (see 2.1), care had to be taken to find a metadata schema that is compatible with all the data and scientific domains included and covers the most important terms. The majority of the RDIs use Schema.org and the others are compatible and capable of converting their metadata into this schema. Schema.org offers a widely used standardized tagging vocabulary. However, its general-purpose nature presents limitations for the complex and diverse data needs of the agrosystems community. Schema.org lacks specialized profiles for scientific data domains, hindering seamless integration across disciplines due to the use of varying format standards and vocabularies.

For this purpose, it is possible to expand Schema.org with more specific extensions and profiles. Leveraging Schema.org as a foundation, Bioschemas provides domain-specific extensions tailored to the life sciences. This community-driven effort establishes standardized markup for biological data. Bioschemas, through collaboration between researchers, developers, and life science organizations, defines and expands schemas for various biological data types.

To complement the efforts of the middleware in facilitating a centralized access to RDI contents, FAIRagro simultaneously works on extending the vocabulary already available via Schema.org/Bioschemas towards requirements of its community. For this reason, an agrosystem-focused Bioschemas working group was founded that develops new types and properties to further describe agrosystem resources. By analyzing the markup of published datasets and comparing what search queries to find them are already possible with future needs of e.g. FAIRagro’s scientific use cases, a user-driven extension will enable RDIs already providing markup in Schema.org/Bioschemas to increase the findability of their resources.

#### 2.3.2 Middleware concepts in life sciences

This section describes three different archetypes, which have been investigated to distill suitable concepts and technologies for implementing the middleware infrastructure. The aim was to evaluate whether an existing approach or a single component of it can be used or adapted.

##### 2.3.2.1 Beacon

**Figure 1:**
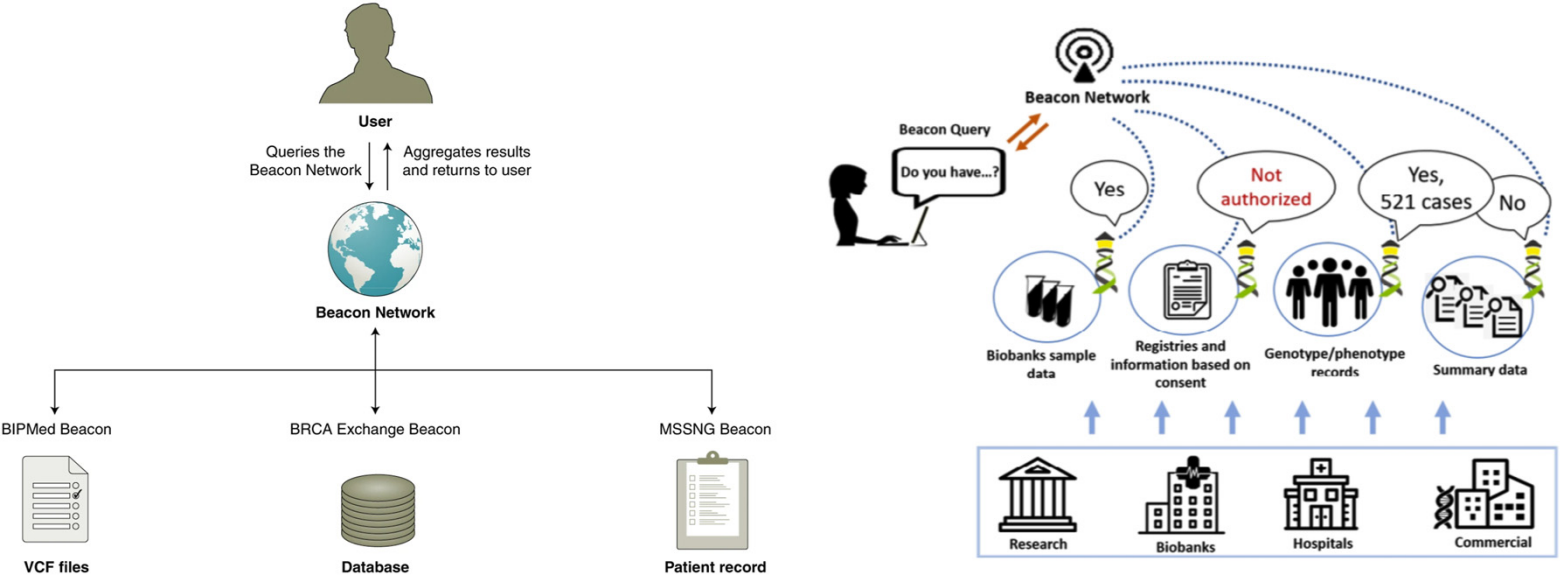
The Beacon concept. Left figure taken from [50]. Right figure taken from [51].

The Beacon Project represents a significant advancement in facilitating controlled access to genomic and phenoclinic data. Developed under the auspices of the Global Alliance for Genomics and Health (GA4GH), Beacon utilizes a standardized protocol to enable efficient discovery of genetic variants across a distributed network of data repositories [49], shown on the left side of Figure 1. This approach prioritizes secure data sharing by enabling researchers to identify datasets containing specific variants of interest without granting access to the underlying raw genomic data. This safeguards patient privacy while promoting collaboration and accelerating research progress.

Beacon achieves this through a robust technical framework. The infrastructure uses a network of individual Beacon resources, each adhering to the Beacon API, a standardized interface that governs data discovery functionalities. Researchers submit queries through this API, specifying the targeted genetic variant. Beacon resources then respond by indicating presence or absence of the variant within their datasets, all without revealing any identifiable patient information, exemplified on Figure 1 (right).

This approach stands in contrast to traditional methods of genomic data sharing, which often involve laborious data transfers and complex access control mechanisms. Beacon enables researchers to quickly identify relevant datasets without the need to directly access the underlying data itself.

Beacon v2 represents the current iteration of the project, offering expanded functionalities beyond basic presence/absence queries. The project website serves as a central hub, providing ongoing updates, detailed technical specifications, and information on working groups actively engaged in the development and refinement of the Beacon standard.

In conclusion Beacon presents numerous strengths, such as its established status within the infrastructure landscape and its successful track record. However, its focus on the genomic and phenoclinic domain could be considered a limitation, potentially restricting its applicability to broader research objectives. Additionally, its reliance on a specific approach for controlling data access through “presence/absence” queries may pose challenges for users seeking more diverse query methods. Nevertheless, the comprehensive documentation of Beacon serves as a valuable resource, providing insights into its secure data exchange mechanisms and efficient standardized APIs. Its open-source nature and CC0 licensing further enhance accessibility but could limit adaptability due to technological dependencies. Moreover, while Beacon’s robust technical framework and Beacon API are strengths, they also necessitate widespread user adoption for optimal functionality, which may pose a barrier. Finally, the limited domain focus on clinical studies could be seen as a constraint, potentially limiting its relevance for research objectives outside this scope.

##### 2.3.2.2 OmicsDI

**Figure 2:**
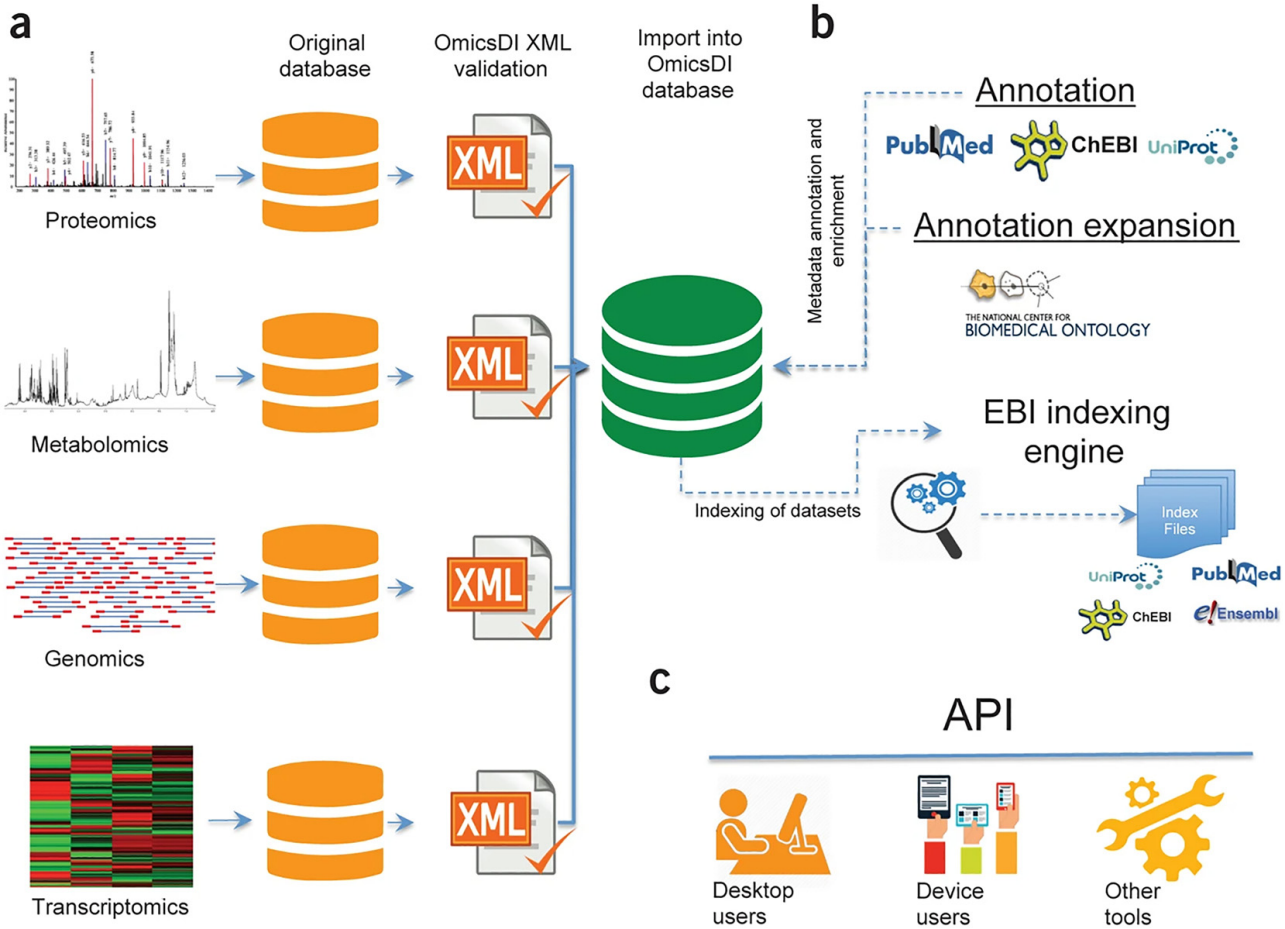
The OmicsDI concept. (a) Omics data from public repositories is converted to a unified format with all metadata and entities. OmicsDI XML validation ensures data integrity. (b) Public services like UniProt, ChEBI, and PubMed annotate OmicsDI XML files, enriching metadata. The EBI search engine creates indexes with linked resources like PubMed, UniProt, Ensembl, and ChEBI for efficient retrieval. (c) The OmicsDI API provides access to data for various clients, including web interfaces. Figure taken from Ref. [52].

OmicsDI [52], the Omics Discovery Index, is an innovative platform developed under the auspices of ELIXIR, an international effort to harmonize bioinformatics resources across Europe. It serves as an extension to the renowned EBI Search [53], offering enhancements for a specialized subset of EMBL-EBI databases focused on Omics data. Figure 2 exposes how this endeavor addresses a longstanding challenge in the field by providing a unified metadata scheme, thereby streamlining access to diverse biological datasets. OmicsDI operates through a systematic process involving nightly metadata updates from connected databases in its proprietary XML format. These data dumps undergo validation and enrichment, including cross-referencing with other research databases, before integration into the EBI Search Indexer. Consequently, users can access OmicsDI’s comprehensive metadata via both the standard EBI search interface and its dedicated web interface and API, which offer additional Omics-specific functionalities.

While OmicsDI demonstrates promise, there are certain limitations that hinder its suitability for our research goals. Specifically, OmicsDI lacks the ability to incorporate agro-system specific or geospatial metadata. To address this limitation, we could leverage general-purpose attributes, but given our greenfield approach, it is not desirable to implement workarounds at the outset. Additionally, OmicsDI relies solely on text-based search queries, which may not be suitable for numerical or geospatial data-based searches and it does not provide the capability to output FDOs. The lack of comprehensive documentation necessitates close collaboration with the OmicsDI developers, whose willingness to provide assistance is uncertain. Furthermore, the architectural dependencies of OmicsDI pose challenges, requiring collaboration with EMBL-EBI, which may result in legal entanglements.

##### 2.3.2.3 Annotated research context (ARC)

**Figure 3:**
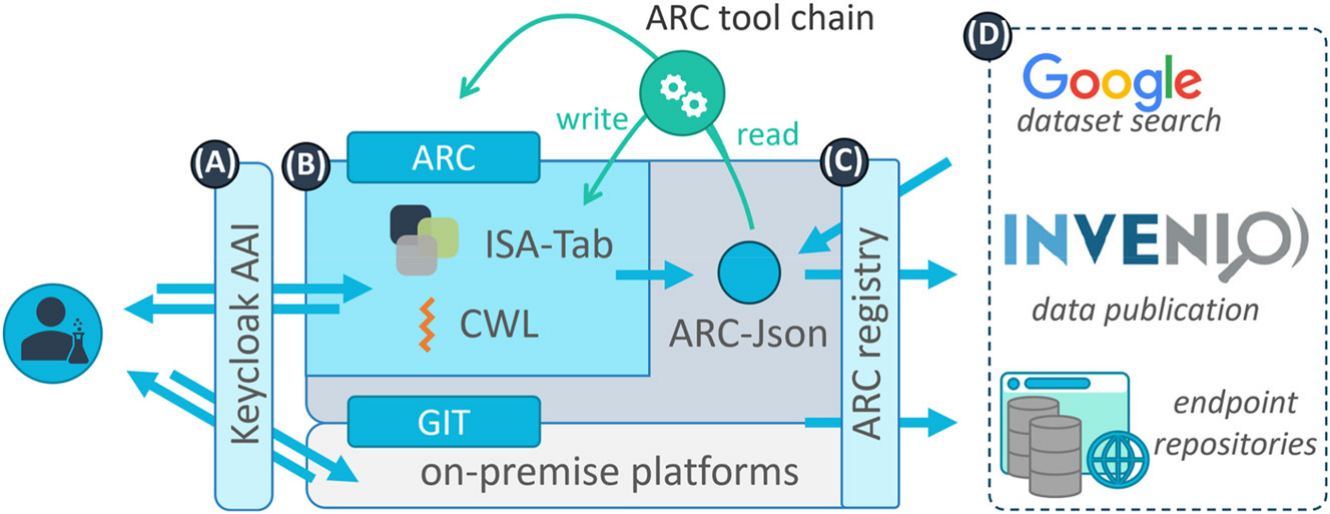
Schematic overview of the PLANTdataHUB architecture. This graphic was taken from [54] and shows the four major components of the PLANTdataHUB infrastructure. A Keycloak instance (A) takes care about the authentication and authorization for creating and accessing ARCs (B) within the GitLab-based ARC-registry (C). Via a connected InvenioRDM instance, ARCs can be published by assigning a persistent DOI.

As the FAIRagro consortium is part of the larger NFDI we also looked for relevant approaches and potential cooperation within the network of consortia. One very promising concept is that of the Annotated Research Context (ARC), which was developed and used by the DataPLANT consortium [54]. ARCs are FAIR Digital Objects (FDOs), which represent digital entities that are describing the actual data and metadata in association with mechanisms for creating and (re-)using them following the FAIR data principles. They are self-contained and machine-actionable data containers [16]. To enable collaboration and a continuous data curation the ARC container was combined with the widely used and in software development established Git platform to build up a central repository for storing and organizing FDOs. This so-called “PLANTdataHUB”, which general schematic architecture is shown in Figure 3, provides a powerful RDM platform. It is mainly focused on the plant community, but should act as a software-as-a-service blueprint, too [54].

The internal ARC data model is compliant with the ISA model [55] and uses known headers and values derived from ontologies [5], which provides a high flexibility and adaptation for different data domains. By using the Git version control system ARCs can be evolved over time and their history can be tracked back, which is a major difference in contrast to other FDO implementations, such as RO-Crate [46], which were mainly designed to be a final and immutable snapshot of a research dataset. With ARCs the different versions can be created and tracked by using Git commits to create a transparent data provenance, while the machine-actionability is realized by using the common workflow language “CWL” [56] within the ARC container to describe the data applied processes. An additional Keycloak instance [57] acts as authorization and authentication infrastructure (AAI) layer for the PLANTDataHUB. Furthermore, an additional InvenioRDM instance [58] was set-up and connected with the GitLab of the PLANTDataHUB and allows users to publish also their managed ARCs by minting a long-term stable DOI.

Although the original idea behind the ARC concept was to provide a stand-alone fully functional RDM infrastructure for a specific data domain, in this case plant research, the general concept has enormous potential to be applied in other data domains as well. The used ISA model provides a generic data model that is already used in various omics and whose generality promises to be transferable to many other research fields. In addition, the use of the GitLab infrastructure guarantees scalability even with an increasing number of recorded datasets or extensive version histories. In addition, GitLab offers the possibility of defining a wide range of Continuous Integration and Continuous Deployment (CI/CD) processes to establish connections to other infrastructures and thus ensure integration into various technical systems.

#### 2.3.3 Authentication and authorization infrastructure

In order to guarantee data protection and safe access, an AAI system is needed, thus, the investigation of available providers has started. For integration of the FAIRagro middleware into the overall NFDI AAI architecture, the recommendations for an AAI system given by Base4NFDI/IAM4NFDI [59] are taken into account. IAM4NFDI offers community AAI as a service for the NFDI consortia, where four different AAI solutions are proposed. These are Academic ID [60], Didmos [61], Reg-App [62] and Unity [63]. However, there are also other solutions outside of the basic NFDI service e.g. the Life Science Login (LS Login), previously known as ELIXIR AAI [64, 65], which is used e.g. by the de.NBI cloud [66] and is planned to be used by NFDI4Biodiversity [67].

The identification of a suitable AAI system is conducted in a two-stage process. The first step was to get an overview and a comparison of the different AAI solutions. This comprised exploring their websites, searching the documentation, attending workshops, e.g. IAM4NFDI workshops on community AAI or a workshop from NFDI4Biodiversity on LS Login, and establishing contact to the AAI developers or operators, respectively. Further, virtual organizations (VO) at the test instances of the four IAM4NFDI AAI systems were requested in order to evaluate the user management as well as the connection of a Nextcloud instance as an example service.

The result of the AAI comparison was that all of them offer a similar feature set. All are based on the AARC Blueprint Architecture [68], incorporate the eduGAIN system [69] and thus are compatible to the DFN-AAI [70], offer single sign-on functionality, so that user needs to login only once during a browser session, and support the most common authentication protocols (SAML, OpenID connect [OIDC]). Differences between the AAI solutions can be seen in some features due to their configuration options or stage of development. Further they differ with respect to software and support aspects, i.e. the use of open source or commercial software.

Therefore, in the second step, which is currently ongoing, a requirement analysis is conducted to identify the functions that are relevant for the FAIRagro consortium for decision-making. This takes into account the deployment model (multi-tenant system or individual instance), the set of IdPs (academic, community or social authentication providers), services that need to be connected to the AAI, attributes, used to express user information as proposed by IAM4NFDI, as well operational features like multi-factor authentication or account linking.

## 3 Concept of the federated FAIRagro middleware architecture

The following section summarizes the middleware concept for agrosystems data in Germany in the initial FAIRagro project phase. To meet the idea of agile development in a super-complex environment of dozens of RDIs and institutions with diverse operational and resource requirements, we decided to use an incremental approach with step-wise refinement of the middleware concept but support for operational data retrieval services as soon as possible. The results discussed below show the advantage of this approach.

### 3.1 Basic middleware

A technical assessment shows that the majority of the FAIRagro RDIs already expose JSON-LD formed metadata, which either implement Schema.org, Bioschemas, or a proprietary but in general compatible schema. Some are remote retrievable as embedded hidden sections in the landing pages of dedicated datasets and some provided explicitly as remote accessible files. Although the provided metadata comprise mainly technical metadata, i.e. basic information, such as authors and generic keywords, but not semantic details, it is beneficial to harvest them. This provides at least an initial set of minimal metadata for a basic level of RDI-interoperability.

The data harvesting concept consists of two process steps:

a) Discovery service
  In the first step a discovery service identifies a list of all available dataset URLs, e.g. landing pages or REST API endpoints, via RDI-provided sitemaps. The entry point is provided as configuration and results from the assessment done before. Subsequently these URLs are crawled and the JSON-LD metadata is extracted. This process is implemented using *Python* and the *extruct* library [71], which is capable of parsing various types of metadata from HTML pages. The process is executed daily by a scheduler.
b) Harvesting service
  All single metadata files harvested per dataset are integrated into one single JSON-LD file per RDI and versioned persisted in an internal GitHub repository as central access point for the integrated metadata requested by the in parallel developed Search and Inventory Portal and Workflow system SciWIn.

**Figure 4:**
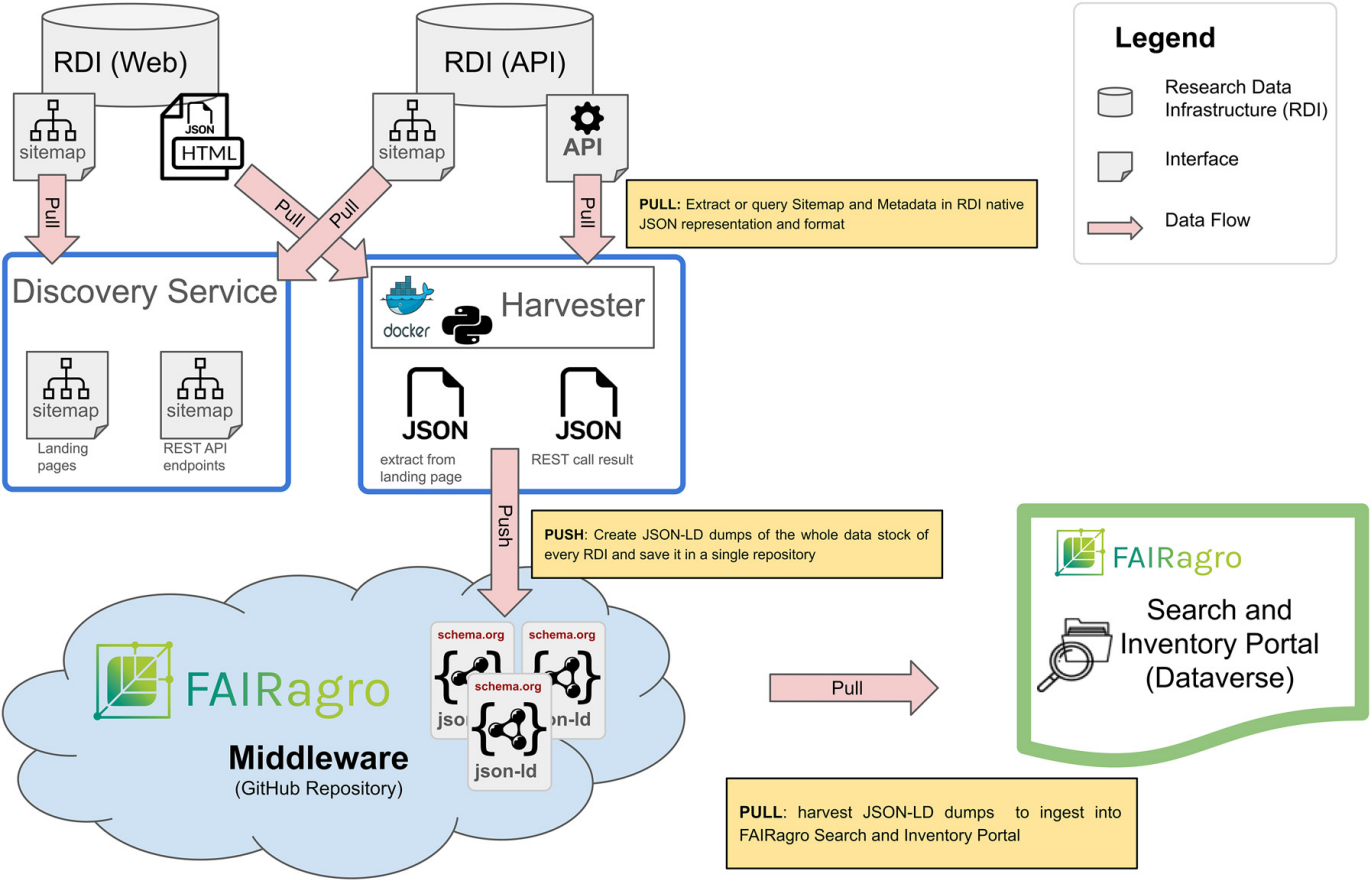
Schematic overview of the FAIRagro basic middleware architecture.

The basic middleware architecture, shown in Figure 4, follows the DevOps-paradigm. It is deployed as docker image [72] on a Kubernetes cluster [73] and provides the first phase nucleus for RDI integration. Its simple architecture and the low implementation effort support a lightweight on-boarding of diverse RDIs and enables agile principles to refine step-wise RDI interfacing, like extension of the integrated agronomic metadata schema, as described in Section 2.2. Furthermore, the downstream services, like the Search and Inventory Portal and the workflow infrastructure SciWIn are able to provide basic services as early as possible.

### 3.2 Extended middleware

In contrast to the metadata-based approach of the basic middleware, the extended middleware implements the requirements for retrieval of integrated, harmonized data across disciplines and RDIs. In this second phase of the middleware implementation, the FDO concept is adapted and the ARC specification as well as the tooling ecosystem developed by the NFDI consortium DataPLANT [74] are reused. This reuse of technology of this, domain-wise closely related NFDI consortium, which was initiated two years in advance to FAIRagro, provides several advantages, which fit very well with the requirements of FAIRagro.

Of central importance is the task of the middleware to enable access to quality assured integrated datasets across domains. An unsupervised tight cross-domain integration of heterogeneous databases is difficult. Rather FDOs as a concept of consistently cross referenced, metadata enriched datasets with compatible data types for the same data domain and a harmonized terminology, units and structures is the alternative. These quality assured integrated datasets are the result of a curation process that starts from the retrieval of stand-alone datasets from the particular RDI, its structural validation and semi-automatic mapping to harmonized units, terminology and data types to manual curation.

**Figure 5:**
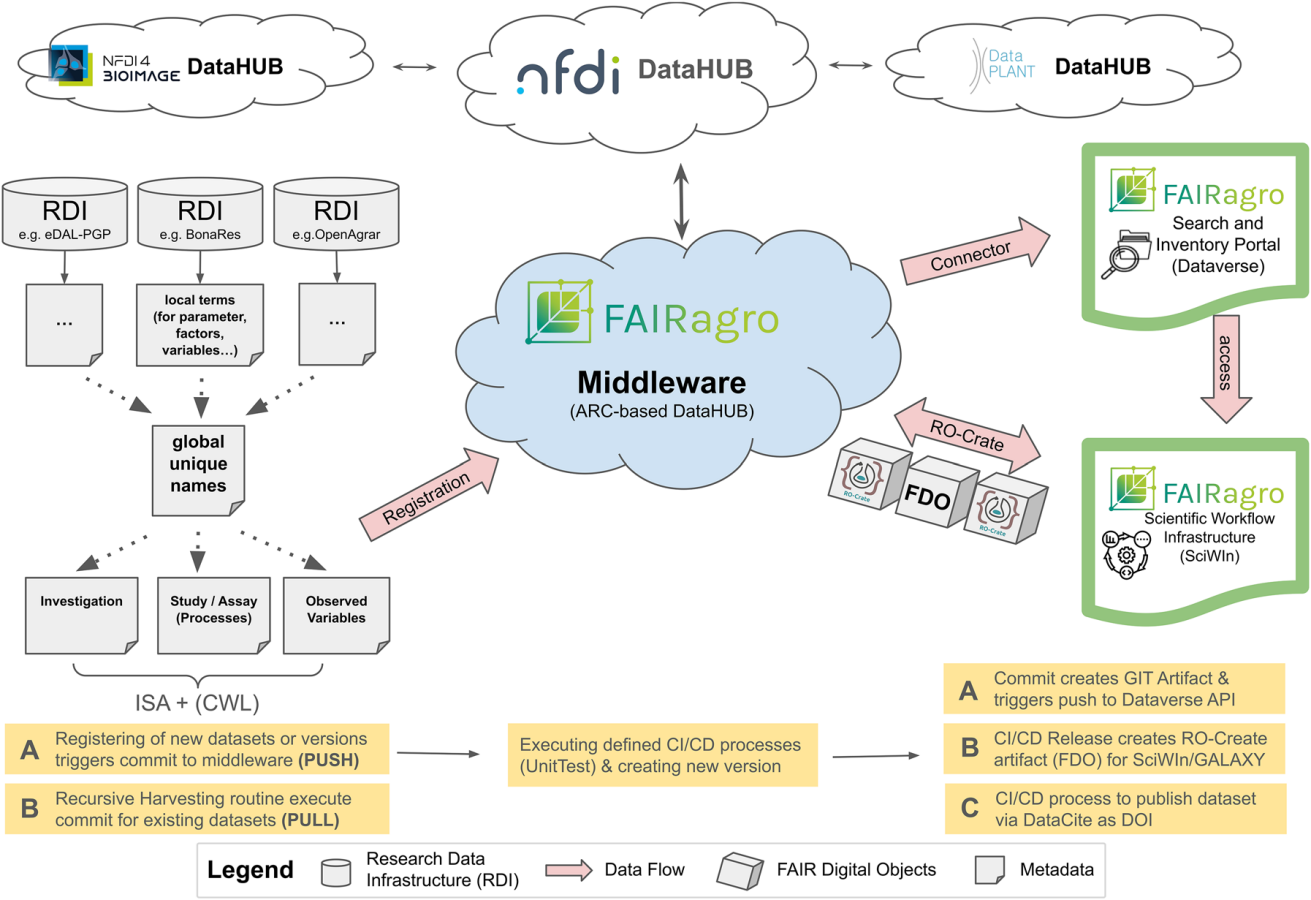
Schematic overview of the FAIRagro extended middleware architecture.

Next requirement is to serve the downstream SciWIn service and to provide RO-Crate formatted FDOs. Since an ARC can be represented as a RO-Crate [75] the SciWIn input and output data format can be based on a modified or extended version of ARC. Therefore, we designed the extended middleware concept shown in Figure 5.

The central component of the extended middleware is an ARC-based GitLab infrastructure, which will be used to register all public datasets provided by the FAIRagro RDIs. Initially, metadata for all remote retrievable datasets will be extracted, e.g. using the basic middleware infrastructure. This will be enriched in the course of the project by a mixed “Push & Pull” strategy, depending on the provided technology stack and the operational processes of the different RDIs. A Push strategy enables RDIs to either actively integrate the construction of ARCs as part of their native data collection and ingestion process to share data records in a central GitLab as FDOs. Alternatively, the advanced middleware supports a passive process that pulls recently created or updated datasets in a regular manner and transforms them in a semi-automated process into raw ARCs, which has to be validated and curated by domain experts afterwards to guarantee data quality and avoid semantic errors.

The major challenge in both strategies is the development of various metadata crosswalks. This ISA based mapping to harmonized metadata concepts for the respective data domains is in general an essential task within the FAIRagro consortium. It has to be mastered across the task areas and use cases. Thus, a close collaboration between use-cases, the RDIs, service developers and experts on standards such as metadata schemas and ontologies, was established early on and will be a continuous process throughout the project. Supporting tools and services by FAIRagro measures for standards in data management, FAIRness and discoverability results in guidance on efficient (re)use of already existing resources, e.g. via the provision of a searchable standards inventory, as well as extension developments of and harmonization mechanisms between relevant standards.

The advantages of this concept are also the scalable data storage backend infrastructure using GitLab and the flexibility and generality of the ISA model used for the FDOs to integrate extremely heterogeneous data records provided in agrosystems science.

The final and enormous important factor for the acceptance of the middleware is the embedded federated AAI to implement data protection and safe access in a trusted manner. We have already started the investigation and assessment of available AAI providers based on requirements arising in FAIRagro from organizational processes to user and role management, granularity of data access permission and governance processes for RDI or FAIRagro wide data use agreements. A multi-tenant AAI infrastructure or a separate instance, respectively, will be set up, to serve the middleware.

## 4 Conclusions

The FAIRagro consortium will develop FAIR compliant infrastructure services for the agrosystems science community and integrate them in the German research data infrastructure service landscape. In this article we have presented a first insight into the development of the FAIRagro middleware infrastructure, which is a central technical building block to connect existing data infrastructures of the FAIRagro community. The challenge lies in the large scientific heterogeneity of the various connected RDIs as well as in the complexity of the technical requirements resulting from the different consumers that need to be connected to the middleware and which build their services on it. While the basic need for a middleware had been already identified during the application phase of the FAIRagro consortium, a comprehensive analysis of specific requirements of the middleware took place during the first phase of the project. This consisted of an extensive investigation of RDIs, which need to be connected, a multi-stage on-boarding process and the evaluation of potential existing software solutions and concepts, which are described within this article. In addition, we have presented a first concrete approach for a technical concept and basic implementation of the middleware to enable the parallel development of consumer services such as the FAIRagro Search and Inventory Portal or the Scientific Workflow Infrastructure (SciWIn) and give an outlook of an extended approach. This extended concept combines various technical standards, software and concepts such as Schema.org, GitLab and FDOs. In order to ensure the complete necessary functionality, we will start in the upcoming project phase with the implementation of the extended middleware for the initial set of RDIs to show that the concept fulfills the defined requirements. Subsequently further RDIs will be step-wise connected into the middleware infrastructure. Another important task will be the further investigation of potential solutions for an Authentication and Authorization Infrastructure and the development of a concept for integrating it into the middleware concept and the interactions between the connected services.
